# Improved Prediction of Preterm Delivery Using Empirical Mode Decomposition Analysis of Uterine Electromyography Signals

**DOI:** 10.1371/journal.pone.0132116

**Published:** 2015-07-10

**Authors:** Peng Ren, Shuxia Yao, Jingxuan Li, Pedro A. Valdes-Sosa, Keith M. Kendrick

**Affiliations:** 1 Key Laboratory for NeuroInformation of Ministry of Education, Center for Information in Biomedicine, University of Electronic Science and Technology of China, Chengdu, China; 2 School of Computer Science and Engineering, Nanjing University of Science and Technology, Nanjing, Jiangsu, China; Duke University, UNITED STATES

## Abstract

Preterm delivery increases the risk of infant mortality and morbidity, and therefore developing reliable methods for predicting its likelihood are of great importance. Previous work using uterine electromyography (EMG) recordings has shown that they may provide a promising and objective way for predicting risk of preterm delivery. However, to date attempts at utilizing computational approaches to achieve sufficient predictive confidence, in terms of area under the curve (AUC) values, have not achieved the high discrimination accuracy that a clinical application requires. In our study, we propose a new analytical approach for assessing the risk of preterm delivery using EMG recordings which firstly employs Empirical Mode Decomposition (EMD) to obtain their Intrinsic Mode Functions (IMF). Next, the entropy values of both instantaneous amplitude and instantaneous frequency of the first ten IMF components are computed in order to derive ratios of these two distinct components as features. Discrimination accuracy of this approach compared to those proposed previously was then calculated using six differently representative classifiers. Finally, three different electrode positions were analyzed for their prediction accuracy of preterm delivery in order to establish which uterine EMG recording location was optimal signal data. Overall, our results show a clear improvement in prediction accuracy of preterm delivery risk compared with previous approaches, achieving an impressive maximum AUC value of 0.986 when using signals from an electrode positioned below the navel. In sum, this provides a promising new method for analyzing uterine EMG signals to permit accurate clinical assessment of preterm delivery risk.

## Introduction

Preterm delivery, or premature birth, is defined as a baby being born at less than 37 weeks gestation, whereas term delivery implies birth occurring at 37–42 weeks[1]. Preterm delivery of babies increases their risk of mortality and morbidity and has a comparatively high average incidence of 5–9% of births in developed countries, although in the USA even higher figures of 12–13% have been reported over the last few decades[2]. In addition, the World Health Organization (WHO) has estimated that about one-third of low birth weight deliveries are caused by preterm delivery [3]. Overall, nearly 10% of neonatal mortality worldwide (500,000 deaths per year) are due to preterm delivery [3]. There are also numerous other health risks associated with preterm neonates including reduced growth, cardiovascular complications, respiratory, gastrointestinal and metabolic (hypoglycemia, hypothyroxinemia) problems, neurodevelopmental (cerebral palsy, mental retardation and sensory impairments) and cognitive dysfunction (impaired attention, executive function, working memory, cognitive flexibility) and major difficulties in academic achievement [4–6]. Finally, preterm birth is costly in terms of healthcare even without taking into consideration the long-term expenses for individuals with subsequent disabilities. In 2007, Institute of Medicine (IOM) reported that the annual cost associated with 550,000 premature babies born each year in the USA could reach up to $26 billion [7]. Thus, any approach which can effectively predict the likely risk of preterm delivery with sufficient reliability to permit appropriate medical intervention will be of great value.

The exact causes of many preterm births are still unresolved, with factors contributing to at least fifty percent of preterm births being unknown[8]. Apart from a number of potential candidates, such as medication, uterine over-distension, preterm premature rupture of membranes (PPROM), intrauterine inflammation, precocious fetal endocrine activation, surgery, ethnicity and lifestyle[9,10], there is still a large amount of uncertainty about their specific risks [11,12]. Hence, it is currently very difficult to make reliable predictions of preterm delivery risk based on them.

Uterine electromyography (EMG) recordings from the abdominal wall of pregnant women represent a noninvasive and economical approach which may provide a sensitive measure of subtle changes in uterine activity indicating risk of preterm delivery [13,14]. Bipolar electrodes spaced 2.5–7cm apart are generally placed over the surface of the abdomen of the pregnant woman [15]. Although monitoring uterine activity using a tocodynamometer (TOCO) or magnetomyogram was also initially thought to be a promising approach for predicting risk of preterm delivery, recent studies have not confirmed this. Studies using uterine EMG recordings have shown that this may be a better approach[16–22].

Many different signal processing techniques have been used to analyze uterine EMG signals. G. Fele-Zorz *et al*. compared various linear and nonlinear signal processing techniques to separate EMG records of term and preterm delivery groups and found that nonlinear ones achieve the best results in discriminating between them [15]. A nonlinear correlation coefficient approach has been shown to improve the utility of uterine EMG signals for clinical purposes [17,18, 21–24]. Diab A *et al*. have also applied nonlinear analysis techniques to classify uterine EMG signals during pregnancy and labor using a time reversibility method [25, 26]. The uterus is comprised of many types of tissue that exhibit nonlinear and dynamic activity [27], thus it seems reasonable to analyze uterine EMG using nonlinear signal processing approaches. Indeed, it is widely proposed that the underlying mechanisms in all biological systems utilize nonlinear rather than linear processes [23, 24, 28, 29].

PhysioBank is a widely used archive of well-characterized digital recordings of physiological signals and related data for the purpose of global biomedical research[30]founded by the Harvard-MIT division of health sciences and technology.The uterine EMG dataset, known as Term-Preterm ElectroHysteroGram (TPEHG) in PhysioBank, was recorded from 1997 to 2006 at the Department of Obstetrics and Gynecology, Medical Centre Ljubljana, Ljubljana [31]. It should be noted that the TPEHG is one of the few good publically available datasets for uterine EMG recordings and is the only one suitable for term and preterm delivery prediction. A number of previous studies have used this TPEHG dataset to develop a reliable preterm delivery prediction. One such study generated moderate maximum and average Area Under the Curve (AUC) values of 0.8900 and 0.7833 respectively for preterm delivery prediction [32], whereas another using a neural network classifier only achieved a best classification accuracy of 72.73% [33]. Considering the importance of this issue, and the necessity of having very high levels of predictive confidence in order to justify possible clinical intervention, a higher classification accuracy is clearly required.

In our study, we also used the uterine TPEHG EMG recording dataset together with a new analytical approach to investigate if we could achieve higher classification accuracy compared with previous methods. Here data from the TPEHG dataset were first preprocessed by Empirical Mode Decomposition (EMD) to extract the number of Intrinsic Mode Functions (IMF) from high to low frequency. Next, the instantaneous amplitude and the instantaneous frequency of each IMF component were computed for further calculations of entropy ratios. In addition, six different classifiers were implemented in order to evaluate the prediction performance of preterm delivery. Finally, in order to evaluate whether a specific electrode position on the abdomen gave the best preterm delivery prediction accuracy, the uterine EMG data from three different positions were analyzed separately.

## Materials and Methods

### Uterine EMG signal records

**Fig 1:**
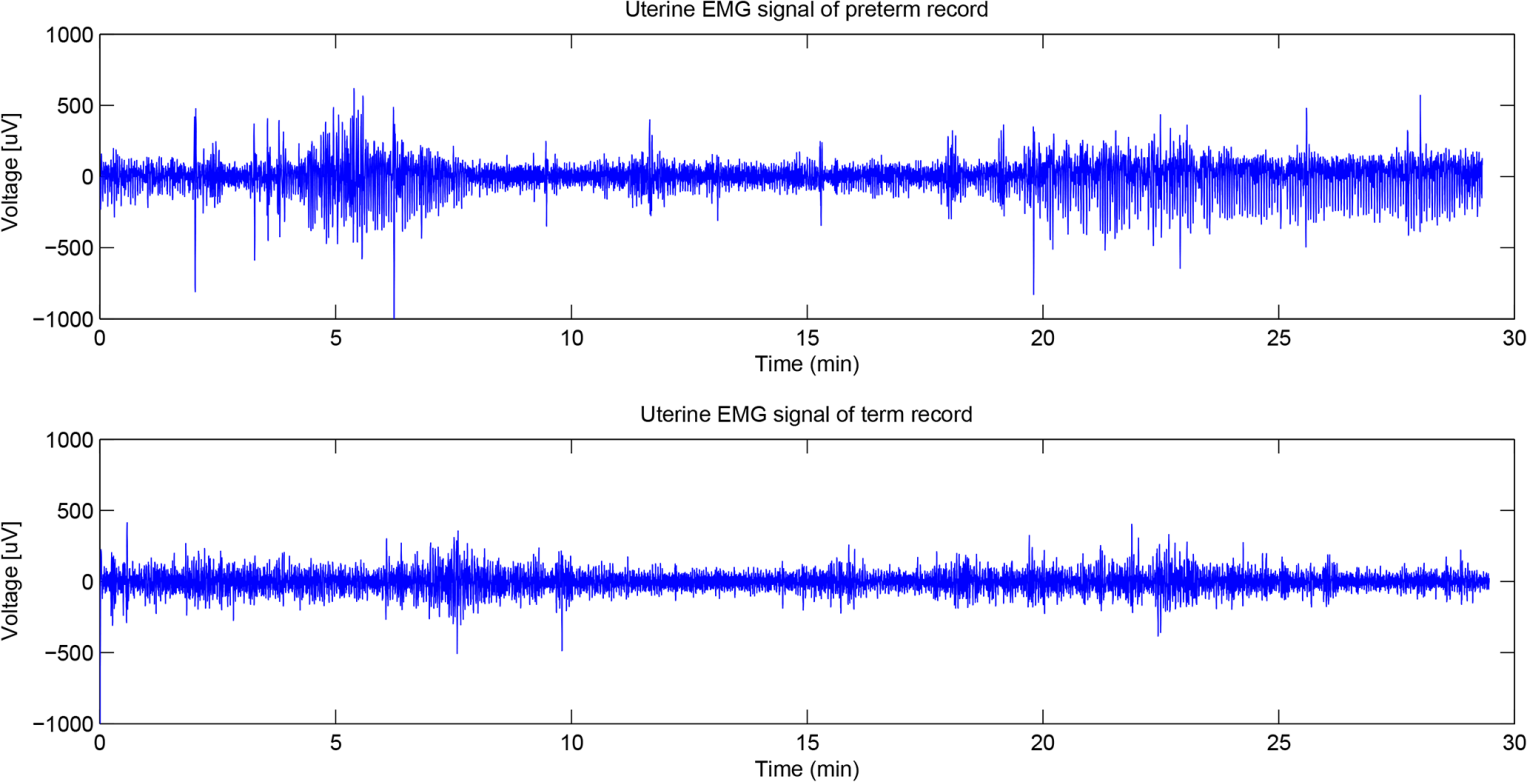
Examples of raw uterine EMG signal records. Data were recorded in the 31th week of gestation. The upper and the lower traces are the examples of preterm delivery (in the 35th week of gestation) and term delivery (in the 40th week of gestation) records respectively.

In our study we utilized the TPEHG dataset in PhysioBank[31] for analysis, which contains a total of 300 individual records (300 pregnancies, one record per pregnancy was recorded). Each record lasts about 30 minutes and contains data from 3 recording channels (four electrodes were attached to the abdominal surface of the pregnant women in two pairs symmetrically under and above the navel, spaced 7cm apart.). The first, second and third channel recordings were measured between the top two electrodes, the left two electrodes and the lower two electrodes respectively. In these 300 uterine EMG data records, 262 records are from pregnancies where the deliveries were term (pregnancy duration = >37 weeks) and 38 records are from pregnancies which ended prematurely (pregnancy duration<37 weeks). The average time of data recording across the 300 pregnant women was 26.68 weeks of gestation. The sampling frequency in the TPEHG dataset is 20 Hz. Fig 1 shows examples of raw uterine EMG signals. In order to explore the approaches which could better classify these term and preterm delivery uterine EMG records we first carried out a preprocessing filtering step. Frequency distributions of uterine EMG signals are totally different from the traditionally analyzed musculoskeletal EMG signals and previous research has suggested that the frequency band from 0.3 to 3 Hz contains the most useful information and excludes most artifacts due to motion, respiration, and cardiac signals (in the TPEHG dataset, the filtered uterine EMG signals are available with various frequency bands, such as 0.08–4 Hz (Butterworth digital filter), 0.3–4Hz and 0.3–3Hz) [15], [32]. A previous study chose the data in channel 3 (the two electrodes below the navel) for analysis to achieve a relatively satisfactory discrimination accuracy[32]. In our study, we also used recorded data from channel 3 with a 0.3–3Hz frequency distribution first. Next we made a comparison between these recordings from the third channel and those from the other two channels from different positions on the abdominal surface.

### Empirical mode decomposition

EMD is a procedure for decomposing a signal into a finite and often-small number of IMFs [33, 34]. The EMD method is self-adaptive and based on the local characteristic time scale of the data. It is therefore highly efficient for processing non-linear, non-stationary and time varying data [35]. IMFs are a series of data sequences with different eigenscales. Each IMF function has the same number of extrema and zero crossings, with its envelopes being symmetric with regard to zero[36]. The process of extracting an IMF is known as sifting and the detailed procedures are as follows: (1) identification of the local extrema of *x(t)*; (2) interpolation of the set of maximal and minimal points to obtain an upper envelop *x*_*u*_ *(t)* and a lower envelop *x*_*l*_ *(t)*, respectively, and their average *m(t) = [x*_*u*_ *(t)+x*_*l*_ *(t)]/2*; (3) subtraction of the average from the original to get *d(t) = x(t)-m(t)*, and (4) repetition of step (1–3) until *d(t)* satisfies the two conditions for being an IMF. Once an IMF is generated, the residual signal *r(t) = x(t)-IMF*_*1*_ *(t)* is regarded as the original signal, and steps (1–4) are repeated to yield the second IMF, and so on. The procedure is complete when the amplitude of the residue falls below a pre-determined small value so that further sifting would not yield any useful components. These features guarantee the computation of a finite number of IMFs within a finite number of iterations. The outcome of the EMD procedure is the following decomposition of the original signal:

$$x(t)=\sum_{i=1}^{n}IMF_{i}(t)+r(t)$$ (1)

where *IMF*_*i*_ *(t)* is the ith IMF, n is the total number of IMFs, and *r(t)* is the final residue that has near zero.

**Fig 2:**
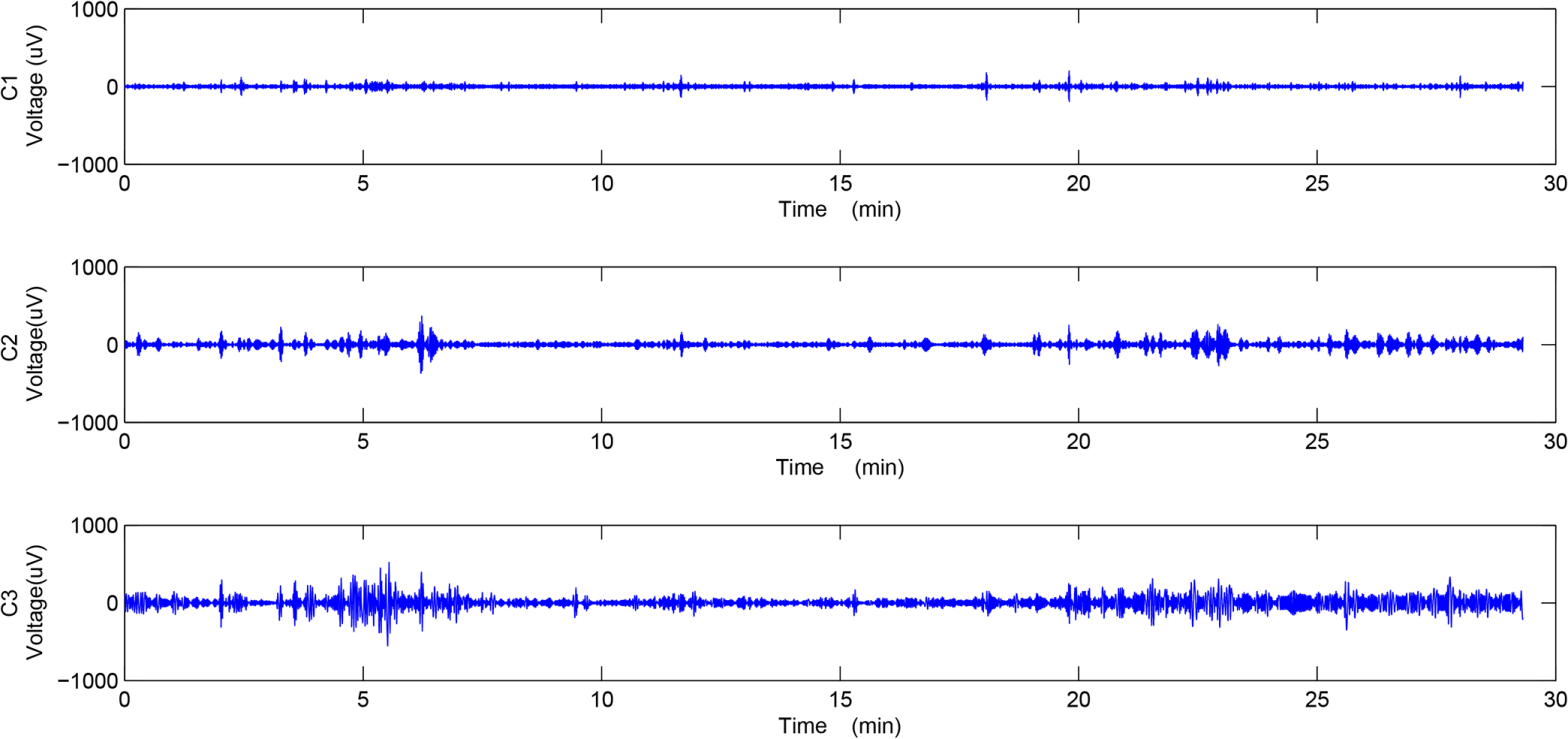
Empirical mode decomposition of a preterm delivery record. The top (C1), middle (C2) and bottom (C3) plots show the first three components of the IMFs obtained by EMD.

The EMD method decomposes the signal into a number of IMFs, which are sequentially ranked from the high to the low frequency components, and then finally the residue. In our study, we implemented the G-Rilling EMD toolbox to achieve IMFs[37].The first ten components were selected for further analysis. Fig 2 shows the first three IMFs of the preterm delivery record displayed in Fig 1.

Through the EMD method, each of the components can be expressed by means of a Hilbert transform in order to calculate the instantaneous amplitude and the instantaneous frequency of that component[38–40]. The formula for the Hilbert transform is given below:

$$y(t)=\frac{1}{\pi }P\int_{-\infty }^{\infty }\frac{IMF(\tau )}{t-\tau }d\tau$$ (2)

where *P* is the Cauchy principal value. The analytic signal *z(t)* can be expressed as the composition of *IMF(t)* and *y(t)*:

z(t)=IMF(t)+iy(t) (3)

The time varying or instantaneous amplitude *a(t)* and phase *θ(t)* can be defined as:

$$a(t)=\sqrt{IMF^{2}(t)+y^{2}(t)}$$ (4)

$$\theta (t)=arctan\left(\frac{y(t)}{IMF(t)}\right)$$ (5)

The instantaneous frequency can be derived from (5) and the equation is given below:

$$\omega (t)=\frac{d\;\theta (t)}{dt}$$ (6)

**Fig 3:**
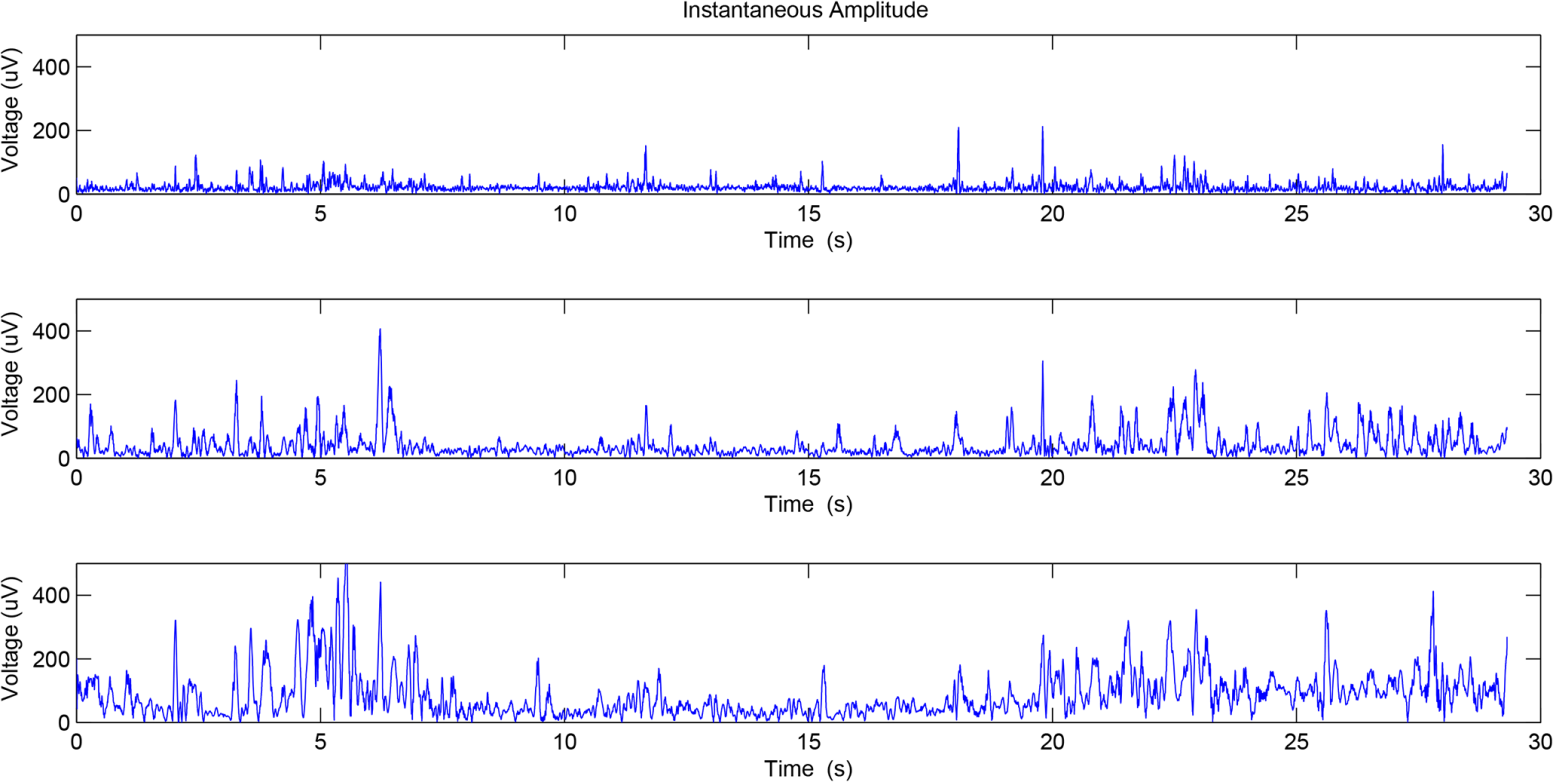
The instantaneous amplitude of the first three components obtained by EMD.

**Fig 4:**
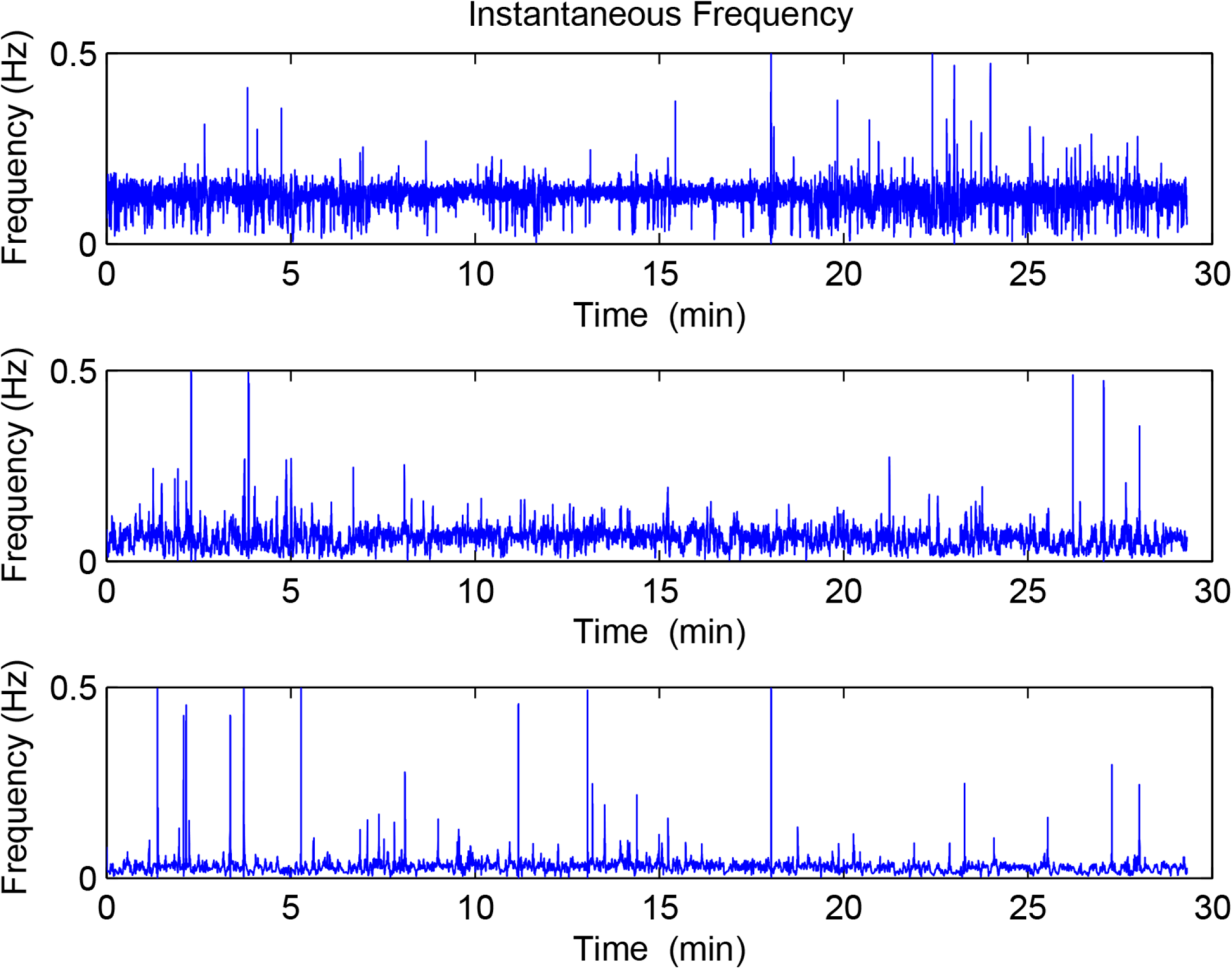
The instantaneous frequency of the first three components obtained by EMD.

Figs 3 and 4 show the instantaneous amplitude and instantaneous frequency of the first three IMFs of the preterm delivery record displayed in Fig 2.

Entropy is one of the most widely used complexity measures in biomedical signal analysis [41]. In our study Shannon entropy was used to calculate the average uncertainty or unpredictability of the instantaneous amplitude and the instantaneous frequency of the first ten IMF components of the uterine EMG signals obtained by EMD. In this way twenty entropy values can be derived from each EMG recording (ten entropy values for evaluating instantaneous amplitude and ten entropy values for evaluating instantaneous frequency, respectively).

Intrinsic relations between IMFs have been shown to be important for exploring a variety of biomedical information. The instantaneous amplitude ratio of the third and the first component of the IMFs of Heart Rate Variability (HRV) can, for example, be calculated as a function of time to detect postural changes in a subject from being seated to standing[42]. In addition, the instantaneous frequency seems to be a good index for separating and tracking HRV rhythms. Furthermore, an algorithm has been developed which can automatically detect epileptic spikes in Electroencephalogram (EEG) recordings by comparing the instantaneous amplitudes of the different IMF components [43]. Hence, in our study the entropy ratios of the instantaneous amplitude and the instantaneous frequency of each two IMFs of the uterine EMG signals were calculated for the purpose of exploring the intrinsic relations between IMFs, given by Eqs (7) and (8).

$$R_{amplitude}(i,j)=\frac{E_{amplitude}(i)}{E_{amplitude}(j)}\;\left(\text{i},\;\text{j}=1,\ldots ,10\right)$$ (7)

$$R_{frequency}(i,j)=\frac{E_{frequency}(i)}{E_{frequency}(j)}\;\left(\text{i},\;\text{j}=1,\ldots ,10\right)$$ (8)

Here *E*_*amplitude*_ *(i)* and *E*_*frequency*_ *(i)*denote the entropy values of the instantaneous amplitude and the instantaneous frequency of ith component of IMFs of the uterine EMG signals, respectively. *R*_*amplitude*_ *(i*,*j)* and *R*_*frequency*_ *(i*,*j)*denote the entropy ratios of the instantaneous amplitude and the instantaneous frequency of ith and jth component of IMFs of the uterine EMG signals, respectively.

### Preterm delivery prediction

In the TPEHG dataset, there are 38 positive values (preterm delivery, minority class) and 262 negative values (term delivery, majority class). Hence, the prior probabilities of the two class datasets are not equal, which would result in classifiers being more sensitive in detecting the majority class than the minority one. There are two approaches to address problems of unbalanced datasets: under sampling and over sampling[44]. If the under sampling method is utilized, 224 pregnancy records will be removed in order to make the number of records of the majority class equal to the minor one. Alternatively, with an over sampling approach additional data is generated in the minority class in order to make the two class datasets balanced. Generally, the over-sampling approach avoids problems of data loss and provides more accurate results than under-sampling methods in terms of the area under the ROC curve (AUC) [44].

A previous study has applied the synthetic minority over-sampling technique (SMOTE) to classify the records of preterm and term delivery groups in the TPEHG dataset [32]. In our study, the same SMOTE approach was also used. The main idea of SMOTE is to form new minority class examples by interpolating between several minority class examples that lie together. Thus, the overfitting problem is avoided and causes the decision boundaries for the minority class to spread further into the majority class space [45]. New samples are generated in the following way: (1) Take the difference between the feature vector (minority class example) under consideration and its nearest neighbor (2) Multiply this difference by a random number between 0 and 1, and add it to the feature vector under consideration. This causes the selection of a random point along the line segment between two specific features. This approach effectively forces the decision region of the minority class to become more general. Thus, it provides more related minority class samples to learn from and allows a learner to form broader decision regions, leading to greater coverage of the minority class. As such, SMOTE has become adopted as the standard method for resolving the unbalanced dataset issue and has been widely used on clinical datasets in cancer and neuroscience research where numbers of patients and controls are often unbalanced[46–48].

Machine learning techniques (specifically, the classification) were utilized to determine the class membership of each dataset (262 preterm/262 term). Six different representative classifiers were implemented using the Waikato Environment for Knowledge Analysis (WEKA) software[49].Support vector machine (SVM) is an associated learning algorithm which is a model for mapping examples of separate categories divided by a margin that is as wide as possible. The idea behind SVM is to map the input data to some high dimensional space using a kernel function where the data can be separated, thus providing good classification (or regression) performance[49], [50]. In our study a polynomial kernel was implemented. Random forests (RF) consist of many classification trees. Each tree is based on the values of random vectors which are sampled independently and have the same distributions for all the other trees in the forest. Next, one classification is given by each tree, and then the classification having the most votes over all the trees in the forest is chosen[49,51]. Multilayer perception (MLP) is a commonly used feed-forward artificial neural network model which consists of multiple layers of nodes where each layer is fully connected to the next one. Backpropagation is implemented in the data training part by changing the layer connection weights through the least mean squares (LMS) algorithm[49,52]. AdaBoost (AB) is a machine learning meta-algorithm which can combine many other types of learning algorithms to improve their performance. AdaBoost is an adaptive classifier which changes instance weights such that the learning algorithm can be forced to focus on a particular set of instances that are previously misclassified [49,53]. In our study the basic classifier used in AB is the decision tree. Bayesian network (BN) is a probabilistic graphical model, which uses graphical structures to represent knowledge about an uncertain domain. Each node in the graph represents a random variable, while the edges between the nodes represent probabilistic dependencies among the corresponding random variables[49,54]. Simple logistic regression (SLR) is a classifier for building linear logistical models[49].

In our study a 10-fold cross validation method was employed. This is a widely used model validation technique for assessing the generalization capability of a classifier, where only one sampled dataset is available. The original dataset was first divided into ten equal subsets, and then one subset was tested using the classifier trained on the remaining nine subsets. This procedure was repeated until every subset had been used once for testing. The overall accuracy of the classifier is based on the average performance over the ten classification runs. Area under the curve (AUC) was then calculated for each classification method with a larger AUC value indicating a greater discrimination potential[55].

## Results

**Fig 5:**
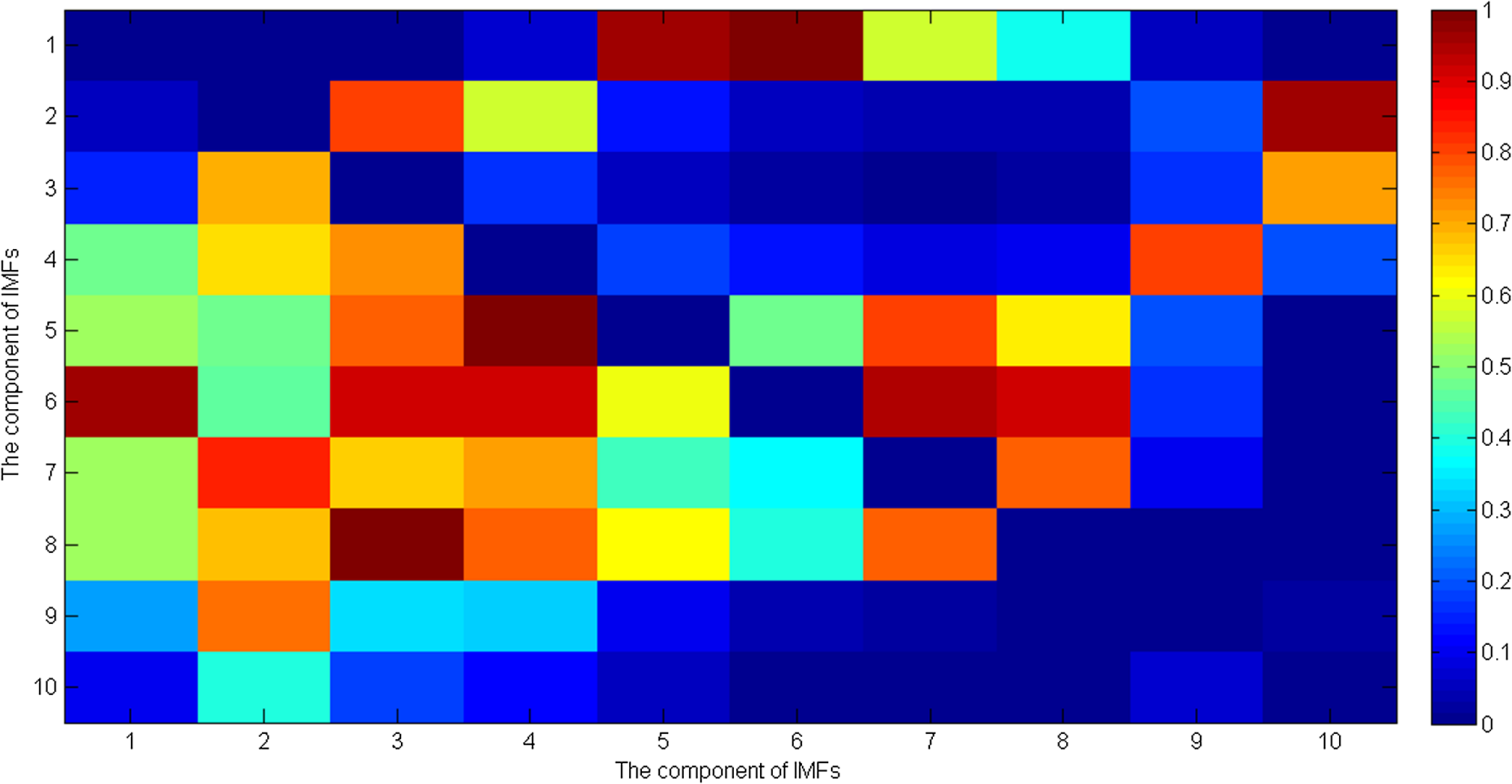
*p* values of Student’s *t* test of the entropy ratios of the instantaneous amplitude of ith and jth component of IMFs of the uterine EMG signals.

**Fig 6:**
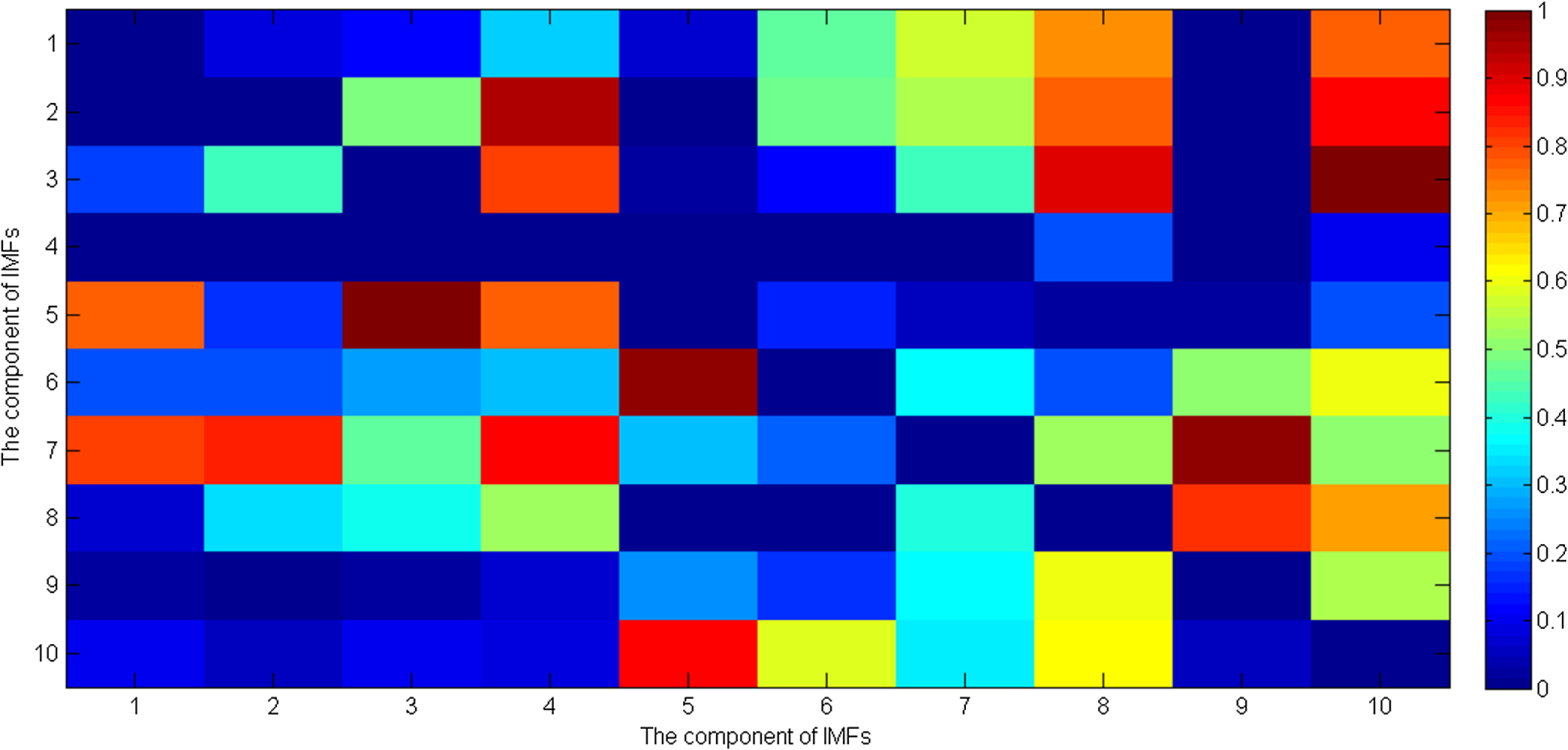
*p* values of Student’s *t* test of the entropy ratios of the instantaneous frequency of ith and jth component of IMFs of the uterine EMG signals.

Independent *t* tests were carried out on measures of *R*_*amplitude*_ and *R*_*frequency*_ between all term (262 pregnant women) and preterm (38 pregnant women) subjects’ uterine EMG records. Fig 5 shows the p values of Student’s *t* test of the entropy ratios of the instantaneous amplitude of ith and jth component of IMFs of the uterine EMG signals. Fig 6 shows the p values for Student’s *t* test analysis of the entropy ratios of the instantaneous frequency of the ith and jth components of IMFs for the uterine EMG signals. It should be noted that the p values across the diagonal in Figs 5 and 6 were not considered in the analysis and so in Fig 5 the number of significant values(p<0.05) is 22 (out of 90), and in Fig 6 it is 21 (out of 90). All statistical tests were performed using SPSS software (version 17.0 SPSS Inc., Chicago, IL, USA).

**Fig 7:**
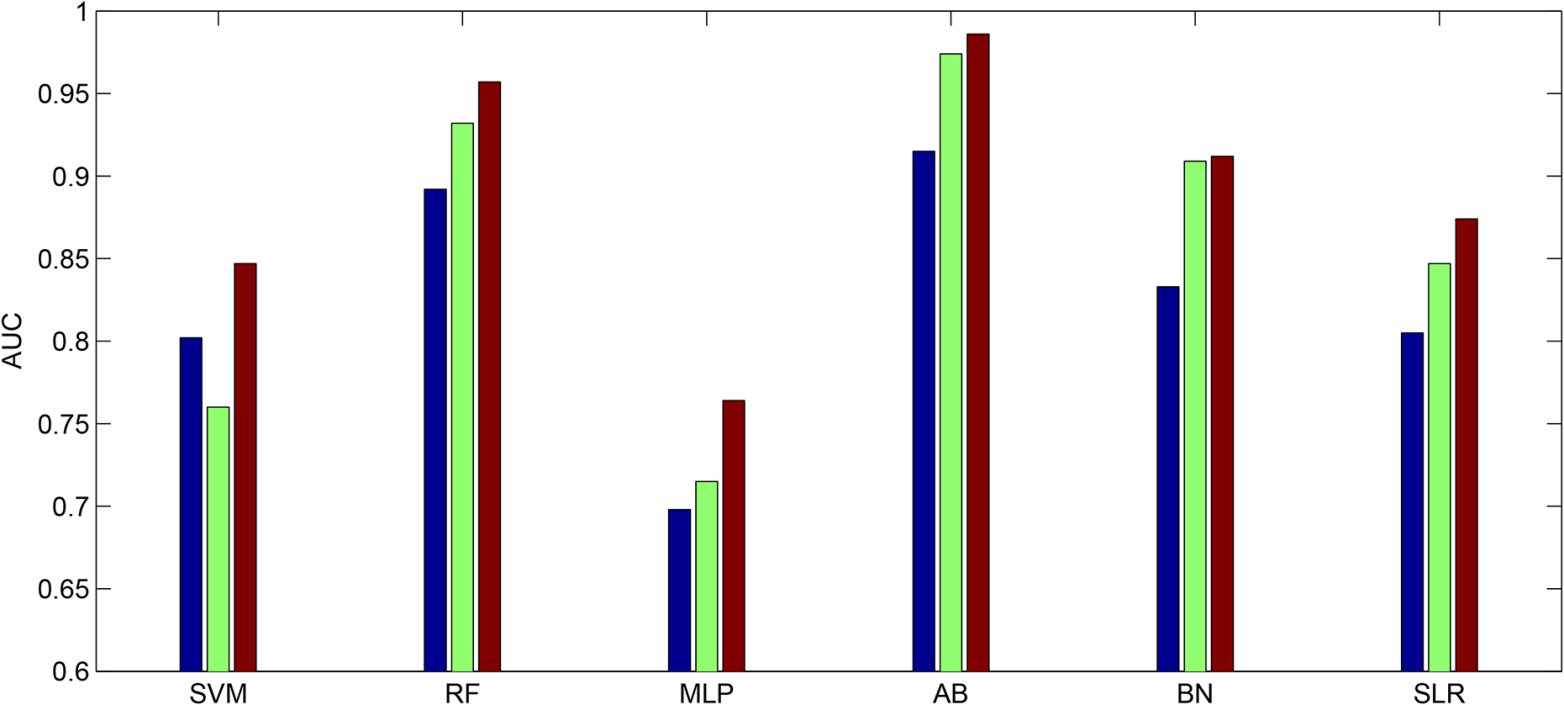
AUC values of the EMD based features derived from channel 1, 2 and 3 of TPEHG dataset for the six classifiers. The blue bar denotes the features extracted from channel 1. The green bar denotes the features extracted from channel 2. The red bar denotes the features extracted from channel 3. (SVM: Support Vector Machine. RF: Random Forests. MLP: Multilayer Perception. AB: AdaBoost. BN: Bayesian Network. SLR: Simple Logistic Regression).

Table 1 shows the classification performances of the extracted features from channel 3 based on both the EMD (180 entropy ratios) and non-EMD methods (P. Ferguset al. used four extracted features together: root mean square, median frequency, peak frequency and sample entropy [30]), respectively. Table 2 displays the ANOVA of the AUC values shown in Table 1 for factor analysis. The results show that the AUC values computed by the EMD method were significantly larger than by the non-EMD method (p = 0.0318). The classifiers have no significant effect for these comparisons (p = 0.1033). In addition, if we compare the AUC values derived from our method and the AUC values given by the previous study [32], the same result was found (the AUC values derived from our methods are significantly larger (p = 0.027)). Finally, Fig 7 shows the AUC values for channels 1, 2 and 3 from the TPEHG dataset for preterm delivery prediction, based on the EMD method and using the same six different classifiers (SVM, RF, MLP, AB, BN and SLR).It can be seen that AUC values for channel 3 are the highest across all the different classifiers.

**Table 1:** Auc of emd and non-emd based features for preterm delivery prediction with 10-fold cross validation.

	EMD	Non-EMD
**Support Vector Machine (SVM)**	0.847	0.540
**Random Forests (RF)**	0.957	0.914
**Multilayer Perception (MLP)**	0.764	0.627
**AdaBoost (AB)**	0.986	0.913
**Bayesian Network (BN)**	0.912	0.842
**Simple Logistic Regression (SLR)**	0.874	0.503

**Table 2:** ANOVA of the AUC Values for Factor Analysis.

Source	SS	df	MS	F	p value
**EMD/Non-EMD**	0.0835	1	0.0835	8.72	0.0318
**Classifiers**	0.16215	5	0.03245	3.39	0.1033
**Error**	0.04787	5	0.00957		
**Total**	0.2937	11			

When we employed principal component analysis (PCA) to reduce the dimensionality of our extracted features (EMD based approach), then 32 new features were obtained (the sum of their percentages of the total variance is above 85%), with an average AUC value of 0.862. If we only select the ***first*** four new features after PCA, an average AUC value of 0.781 is obtained. In contrast, the four extracted features based on non-EMD method only achieve an average AUC value of 0.723. In addition, when we used all the features extracted from three uterine EMG signal channels (180 features per channel, 540 features in total) to classify the preterm and term delivery recordings, this still only achieved an average AUC value of 0.778. However, if we only use the features extracted from channel 3 (180 features) alone, the average AUC value can reach up to 0.89. Thus there appears to be no discrimination advantage in combining features from all 3 channels compared to using recordings from channel 3 alone.

## Discussion

In this paper we proposed a new EMD-based approach to predict the preterm delivery of pregnant women based on the analysis of uterine EMG recordings. Our results have produced the highest discrimination accuracy and AUC values (up to 0.986) so far reported and which may be sufficient for clinical purposes. Previous studies have indicated that uterine EMG signal channel data may be useful in discriminating women at risk of preterm delivery. However, their results showed that the classification performance varied from one channel to another, implying that electrode placement is important. Indeed, studies have indicated that the electrodes below the navel seem to have the largest potential to differentiate the preterm and term delivery groups [15], [30]. In our current study we therefore initially chose recordings from channel 3 (the difference between the two electrodes below the navel) for analysis, although recordings from channels 1 and 2 were also calculated subsequently for comparison. It is apparent from our overall classification performance analysis that channel 3 is indeed much better than channels 1 and 2, as illustrated in Fig 7 and that there is no discrimination advantage in combining features from all three channels. Hence, in future studies uterine EMG recordings from electrodes below the navel are likely to provide the greatest prediction for risk of preterm delivery. Indeed, two other recent studies have also both used electrodes underneath the navel for uterine EMG signal recording, which further supports our suggestion [13, 14].

For processing the signals from uterine EMG recordings EMD was employed. The main conceptual innovations here were the introduction of IMFs based on the local properties of two different components, the instantaneous amplitude and the instantaneous frequency. Uterine EMG recordings essentially comprise non-stationary and non-linear signals, so it is very appropriate to use EMD to analyze them. In our study nearly all the uterine EMG signals are decomposed into 11 or 12 IMFs in total. Because the last IMF may represent the overall signal trend rather than a true IMF only the first 10 IMFs were implemented. The EMD method is similar in some ways to band-pass filtering, although the sub-bands are not predetermined. Thus, many researchers have investigated the properties of these decomposed components (IMFs) separately and found relationships between them. Furthermore, compared with either Fourier or wavelet transforms the EMD is an adaptive process and not limited by the uncertainty principle [56, 57]. In addition, uterine EMG signals from preterm delivery records exhibit higher predictability than those of term delivery records [13]. Hence, in the current study the entropy ratios of the instantaneous amplitude and the instantaneous frequency of each two IMFs of the uterine EMG signals were calculated, respectively. This approach not only includes the average amount of information contained in the signal amplitude and frequency but also reveals the hidden relationship between the two IMFs. Thus, in a previous study by Fele-Zorz *et al*., [15] a minimum p value of 0.011 was found for comparisons between all term and all preterm delivery subjects based on the extracted feature of sample entropy. However, in our more detailed method there are 11 features or entropy ratios for instantaneous amplitude, whose p values are less than 0.011, with a minimum of p = 0.00004681 (see Fig 5).Furthermore, there are 13 features or entropy ratios for instantaneous frequency, whose p values are less than 0.011, with the minimum of p = 0.000048627 (see Fig 6). We infer from this finding that using EMD based entropy ratios as features may be very effective in differentiating between preterm and term delivery uterine EMG recordings. In the classification section we fed our extracted features (entropy ratios), and four extracted features from previous research (root mean square, median frequency, peak frequency, and sample entropy), separately to the same classifiers in order to compare their discrimination accuracy. Because different classification algorithms utilize different techniques to find the relationship between the values of the predictors and those of the target in the model building process, six different types of classifiers were implemented. These classifiers are constructed based on various disciplines including linearity, nonlinearity, probability and graph theory etc. In Table 1 it is obvious that all the classifiers show much better performance over EMD-based features than non-EMD-based ones. In particular, the extracted features based on EMD help increase the AUC values to a mean of 0.89 and a maximum of 0.986. However, the selected features (non-EMD based features) from previous studies can only achieve AUC values with a mean of 0.723 and a maximum of 0.914. Furthermore Table 1 shows that the AUC values derived by EMD method are significantly greater than those derived by non-EMD ones (p = 0.0318).

It should be noted that our findings revealed, the AdaBoost classifier as achieving the highest AUC value of 0.986. AdaBoost is an ensemble learning classifier which has been used, for example, to help business executives seeking advisors whose skills and experience complement rather than duplicate one another[49]. In addition, the AdaBoost classifier can handle weights of instances. The presence of instance weight changes after a classifier’s error calculated by each iteration can force it to concentrate on a particular set of instances, namely those with high weights. Finally, the running time taken to build a model of AdaBoost in Weka for 262 preterm and 262 term subjects is just 4.13s (Intel Core i5-2520M, 2.5GHz). Hence, considering the high AUC value and relatively short running time, AdaBoost may be a promising classifier for preterm delivery prediction for our extracted features.

In comparison with other previous studies using uterine EMG signals to make preterm delivery predictions, some researchers have focused on the classifications of preterm labor and non-labor groups[18]. In this case, preterm delivery refers to women delivering 7 days from the uterine EMG measurement compared to those delivering outside of 7 days (preterm non-labor). Since in our study, the EMD method achieves quite encouraging AUC values for the classification of preterm and term delivery (preterm delivery women: pregnancy duration <37 weeks; term delivery women: pregnancy duration > = 37 weeks), it might also be useful in differentiating between pregnant women with preterm labor and non-labor as well. While M. Lucovnik *et al*[18] implemented power spectrum (PS) and propagation velocity (PV) of uterine EMG to identify true labor and achieved an impressive AUC value of 0.96. They did not control for unbalanced datasets (preterm labor group: n = 20; preterm nonlabor group: n = 68), which might have made this AUC value unreliable. Nevertheless, in view of the significance of the p values they obtained for PS (p = 0.002) and PV (p<0.001), we could in future attempt to combine these two features (PS and PV) together with EMD based ones to try to further enhance preterm delivery prediction.

Some limitations should be acknowledged in our current study. We only employed features based on the EMD method which might not reflect other biomedical characteristics of pregnant women with preterm delivery. In fact, using multiple strategies may further increase the average classification performance of preterm and term delivery, such as combining extracted features derived from different methods [58–62]. In addition, compared with other methods for extracting features for preterm delivery in the literature, the EMD based feature extraction method requires more calculations to be made, although it is still relatively quick and not particularly demanding in terms of computational power.

Overall, our EMD approach has proved to have a significant advantage in identifying women at risk of preterm delivery and has shown that uterine EMG data derived only from two recording electrodes placed below the navel provides the most reliable information. This may already be sufficient for routine clinical purposes, although it is possible that the average classification performance of preterm and term delivery can be increased still further by combining some other kinds of features derived from uterine EMG signals from multiple recorded channels.

## Conclusion

In this paper, we have demonstrated that it is possible to improve analysis of uterine EMG records to discriminate pregnant women at risk of preterm delivery with sufficient accuracy for potential clinical purposes. We have classified preterm and term delivery records based on the entropy ratios of the instantaneous amplitude and the instantaneous frequency of each two IMFs of uterine EMG signals using an EMD approach. Six different classifiers were implemented which revealed a mean AUC value of 0.89 and a maximum value of 0.986. A more detailed analysis of EMG recordings from specific abdominal locations revealed that the position with the strongest discrimination accuracy was the one below the navel. Overall, our study suggests that analysis of uterine EMG signals using the above approach is a very accurate method for discriminating women at risk of preterm delivery and may be important in clinical use.
